# Predictors of thyroid cancer survival in Saudi Arabia: A retrospective 10-year analysis

**DOI:** 10.5339/qmj.2024.44

**Published:** 2024-09-16

**Authors:** Amen Bawazir, Sadeem Alhalafi, Omer Al-Aidaross, Abdulrahman Jazieh, Wasif Ali Khan

**Affiliations:** 1 Department of Basic Medical Sciences, College of Medicine, Almaarefa University, Riyadh, Saudi Arabia; 2 College of Public Health and Health Informatics, King Saud bin Abdul-Aziz University, Riyadh, Saudi Arabia; 3 Specialty business at GSK, Riyadh, Saudi Arabia; 4 Department of Clinical Medical Sciences, College of Medicine, Al Maarefa University, Riyadh, Saudi Arabia; 5 Cincinnati Cancer Advisors, Cincinnati, OH, United States; 6 College of Medicine, Alfaisal University, Riyadh, Saudi Arabia *Email: wkhan@um.edu.sa

**Keywords:** Survival rate, thyroid cancer, papillary thyroid carcinoma, follicular thyroid carcinoma

## Abstract

**Background:**

Thyroid cancer (TC) is becoming more prevalent in Saudi Arabia, currently ranking among the top three cancers affecting women. Despite its rising prevalence, there has been limited assessment of the factors influencing the survival rate (SR) among the Saudi population over an extended period. Therefore, this study aims to address this critical gap in knowledge by identifying the factors affecting the SR of TC, comparing the SR with previous studies, and exploring potential areas for improving the SR of patients.

**Methods:**

A retrospective study analyzed secondary data from patients diagnosed with TC, as recorded in the King Abdulaziz Medical City Cancer Registry in Riyadh, Saudi Arabia, over 10 years from 2009 to 2018.

**Results:**

Of the total 665 TC cases, the mean age at diagnosis was 46.2 years (±SD 16), and most patients were women (78.5%), with the majority being under 50 years old. The most common type of cancer was papillary thyroid carcinoma, comprising 88.6% of all TCs. Over half of the cases were localized to one of the lobes of the thyroid gland, with almost equal frequency between the two lobes. The 5-year SR of localized papillary thyroid carcinoma reached 96.5%, in contrast to the extremely low SR of anaplastic thyroid carcinoma, where most patients died within a few months of the diagnosis. Factors such as morphology, tumor extension, male gender, and age at diagnosis significantly impacted patient survival, as analyzed by the Kaplan–Meier test (*p* < 0.001). Compared to other types of cancer, those with anaplastic thyroid carcinoma had a lower SR.

**Conclusion:**

The SR of TC patients is predicted by factors such as their age, morphological type, and the presence of distant metastasis.

## 1. Introduction

Cancer originating from the thyroid gland ranks as the second most common cancer among females in Saudi Arabia (SA), following breast cancer, with an age-standardized (AS) incidence rate of 11.3 per 100,000 and an AS mortality rate of 1.3 per 100,000 population, as reported by the International Agency for Research on Cancer (IARC).^1^ Over the past 30 years (1999–2019), the incidence of thyroid cancer (TC) in females has surged by 15-fold. In males, TC is the eighth most common cancer in SA, experiencing a 22-fold increase during the same period.^2^ A separate study highlighted TC as exhibiting the greatest increase in incidence in SA between 1990 and 2016.^3,4^ Overall, there is a gradual rise in the cancer burden within the Saudi population year by year. Despite substantial investments in its healthcare sector over the past two decades, leading to advanced and quality healthcare accessibility for its growing population, breast, colorectal, and TC are the top three common cancers in women in Saudi Arabia, according to the IARC, GLOBOCAN 2022 report.^5^ With early diagnosis and appropriate treatment of TC, it is anticipated that there should be a decreasing trend in mortality rates and a gradual improvement in the survival rate (SR) among diagnosed patients. Although breast cancer remains the most frequent type of cancer among Saudi females, the Saudi Cancer Registry (SCR) reported 1045 newly diagnosed TC cases among females in 2018, representing 8.4% of all cancer cases among Saudi females. The eastern region, followed by the Riyadh (central) region, reported the highest number of TC cases in the country.^6^

The National Cancer Institute (NCI) in the United States defines the 5-year SR as the percentage of people alive 5 years after they were diagnosed or started treatment for a disease.^7^ In Western countries like the United States, the United Kingdom, and Australia, the SR for TC has been gradually improving due to the ready availability of screening methods, early diagnosis, and advanced management facilities.^8–12^

While most studies from SA have explored the incidence and prognosis related to TC diagnosis and management, there has been limited assessment of the factors influencing the SR among the Saudi population over an extended period. Therefore, this study aims to provide a comprehensive understanding of the factors affecting the SR of TC in the central region of SA by analyzing retrospective data.

## 2. Materials and Methods

### 2.1. Study design and setting

A retrospective cohort study analyzed the clinical data of patients with TC recorded in the King Abdulaziz Medical City Cancer Registry (KAMCCR), Riyadh, Saudi Arabia, over 10 years from 2009 to 2018. Cases diagnosed in 2009 were followed up for 5 years until 2014, and this pattern continued for subsequent years. The study investigated the association between socio-demographic factors, disease stage, histopathologic subtype of TC, and their impact on SRs.

### 2.2. Participants

All patients diagnosed with TC from January 1, 2009, to December 31, 2018, were included, while cases lacking essential data such as age, gender, and cancer type were excluded. For ethical considerations, the analysis of the study was performed after receiving the IRB approval on June 23, 2020.

### 2.3. Sample size and sampling technique

According to the principle of the sample size, it is to include all cases diagnosed in the period from January 1, 2009 to December 31, 2019. Therefore, a total of 682 records with TC were targeted in the hospital cancer registry. The data received from the records of 17 patients were not satisfactory and were thus excluded. The remaining sample size was 665 records for the included patients in this study.

### 2.4. Data source and variables

Data retrieved from the KAMCCR included sociodemographic characteristics (age, sex, residency, marital status, and nationality), cancer-related information (diagnosis, histopathologic subtype, and cancer stage), and survival status details to identify survival time, with censoring indicators employed.

Histopathologic subtyping followed the World Health Organization classification of thyroid tumors, and cancer staging adhered to the criteria of the NCI, United States (“Localized": confined to the thyroid gland; “Regional": involving regional lymph nodes; “Distant": representing metastatic tumors).^13,14^ As this study involved retrospective data, it was exempted from the requirement for informed consent, and measures were taken to ensure patient confidentiality. Data quality and completeness were assessed based on the protocol and standardized quality-control procedures from the CONCORD Program for global surveillance of cancer survival.^15^

### 2.5. Statistical analysis

Statistical analysis was performed using Statistical Package for Social Science (Version 23), including descriptive and inferential analyses using proportions and chi-squared tests to assess the association between survival status and sociodemographic parameters, clinicopathologic variables, and stage of cancer. Survival was measured using Kaplan-Meier (log-rank) estimates, and prognostic factors were identified using Cox proportional hazards models to determine hazard associations between variables and the SR of TC patients.

## 3. Results

A total of 665 cases of TC were analyzed. The overall 5-year SR for TC was 88.4%. The mean age at diagnosis was 46.2 years (±SD 16; median age 45 years, ranging from 15 to 96 years), with many cases diagnosed before the age of 50 (36.2%). Females constituted the majority of cases (78.5%). More than half of the cases were localized, involving one lobe of the thyroid gland (53.7%). The predominant morphologic type was papillary thyroid carcinoma (88.6%). However, the SR was lower in males (82%), compared to females (90%). Similarly, the SR in elderly patients aged ≥70 years (64.7%) was lower than in patients younger than 50 years (98.8%). Patients presenting with metastasis (36.4%) had a significantly lower SR compared to those with a localized tumor (96%). Papillary thyroid carcinoma exhibited an excellent SR of 92%, whereas the more aggressive anaplastic thyroid carcinoma showed a dismal SR of 5.3% (Table 1).

The association between survival status and gender, age at diagnosis, tumor morphology, and tumor extension demonstrated statistically significant differences in SR. Gender comparison (male vs. female) (*p* = 0.013), age groups (elderly vs. younger) (*p* < 0.001), tumor morphology (*p* < 0.001), and metastatic vs. localized tumor (*p* < 0.001) were all statistically significant (Table 2). Localized medullary thyroid carcinoma and papillary thyroid carcinoma showed SRs of 100.0% and 96.5%, respectively. In contrast, all cases of anaplastic thyroid carcinoma resulted in a 100% mortality rate. Morphological types such as papillary, follicular, and medullary carcinomas were typically localized or involved regional lymph nodes, whereas anaplastic thyroid carcinoma was aggressive and progressively developed metastasis. A strong association was found between morphology and the local or regional spread of TC (*p* < 0.001). Statistically significant differences were also found between morphology and distant metastasis (*p* value = 0.019).

**Figure 1.**
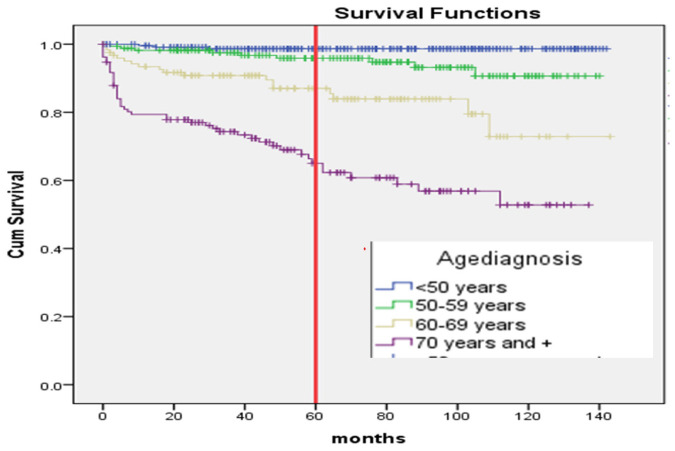
The survival in months for TC patients concerning their age (*p* = < 0.001).

**Figure 2.**
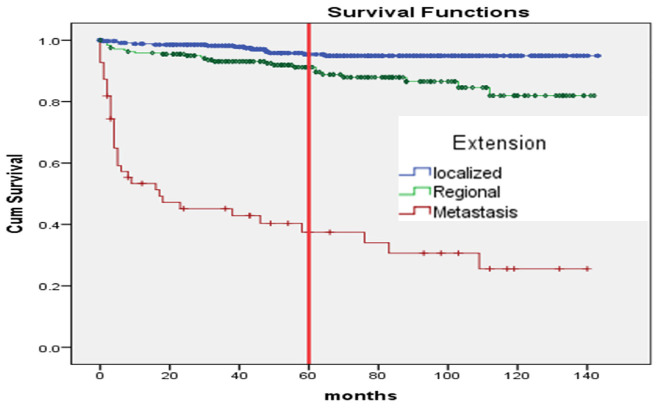
The survival in months for TC patients concerning the stage of cancer (*p* < 0.001).

**Figure 3.**
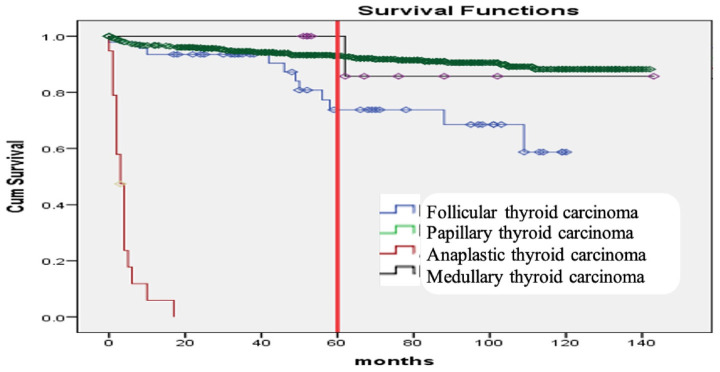
The survival in months for TC patients concerning the histopathologic subtype (*p* < 0.001).

The Kaplan–Meier curve, estimating 5-year survival probabilities, showed significant differences among genders (*p* ≤ 0.008), age groups (> 70 years *vs.* others) (*p* ≤ 0.001) (Figure 1), and patients with distant metastasis compared to local or regional spread (*p* ≤ 0.001) (Figure 2). Anaplastic thyroid carcinoma exhibited a significantly reduced survival compared to other morphological types (*p* < 0.001) (Figure 3).

**Figure 4.**
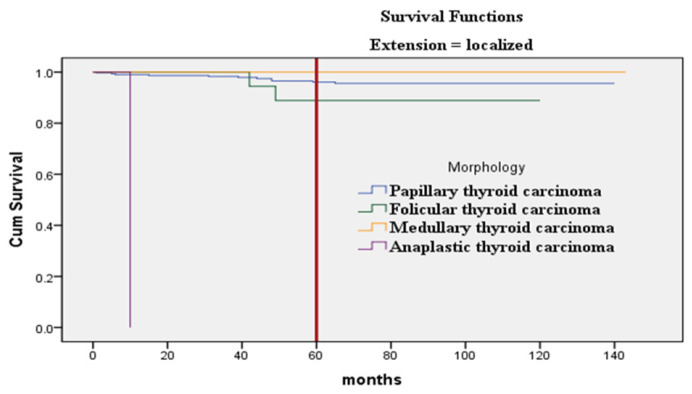
The survival in months of TC patients concerning the histopathologic subtype and “localized” stage (*p* < 0.001).

**Figure 5.**
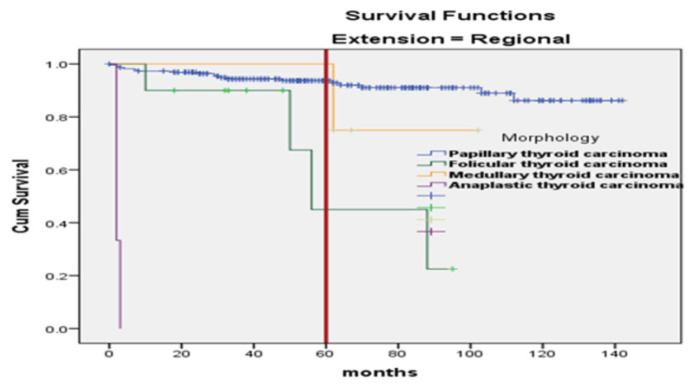
The survival in months of TC patients concerning the histopathologic subtype and “regional” stage (*p* < 0.001).

**Figure 6.**
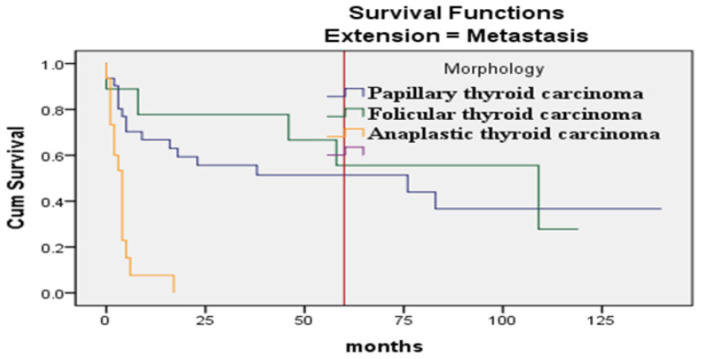
The survival in months of TC patients concerning the histopathologic subtype and “distant” stage (*p* < 0.019).

Additionally, Kaplan–Meier curves for the association of histopathological subtypes of TC with the tumor stage at diagnosis are illustrated in Figures 4–6. Anaplastic thyroid carcinoma, an aggressive cancer, was associated with a significantly lower SR, regardless of tumor localization, involvement of regional sites, or distant spread. Follicular thyroid carcinoma with regional or metastatic disease and papillary thyroid carcinoma with distant metastasis also showed lower SRs. On the contrary, medullary thyroid carcinoma demonstrated a significantly better SR when compared with other morphological types diagnosed with regional or metastatic disease, as no medullary thyroid carcinoma cases with regional or distant metastasis were encountered.

Multivariable Cox proportional hazard models for 5-year disease-free survival were applied to generate hazard ratios related to TC patients’ survival against variables such as gender, age, tumor extension, and morphological types of TC. Patients aged 60 years and above showed a strong association with high mortality (adjusted hazard ratio (AHR) 11.5; 95% confidence interval (CI), 3.3–39.5.9; *p* < 0.001) compared to patients younger than 60 years. Similarly, the comparison of morphological types with tumor extension showed that patients diagnosed with anaplastic thyroid carcinoma with distant metastasis were less likely to survive for 5 years compared to other morphological types with distant metastasis (AHR 6.8; 95% CI, 2.8–16.5; *p* < 0.001 and 7.7; 95% CI 3.7–16.1; *p* < 0.001, respectively) (Table 3).

## 4. Discussion

Our study aligns with the rising trend of TC incidence reported by the latest SCR.^6^ This underscores the need for an in-depth analysis of factors influencing SRs in the Saudi population. While TC generally boasts high SRs compared to other solid tumors, our findings revealed a 5-year SR of 88.4%, which falls below figures reported in developed nations like the United Kingdom (99.2%), Spain (93.1%), the United States (98.0%), and Brazil (98.5%).^16-21^ Interestingly, a previous Saudi Arabian study documented a 94% SR,^22^ suggesting potential regional variations within the country. Additionally, a study from neighboring Oman reported a lower SR of 82.7%.^23^

This observed disparity warrants further exploration. One potential explanation for the lower SR in our study, compared to developed nations and the previous Saudi Arabian research, could be a higher prevalence of advanced-stage diagnoses at presentation, which aligns with the well-documented role of early detection in improving TC survival outcomes.^24^ Multicenter studies encompassing a broader geographical range within SA are essential to elucidate the specific reasons behind the observed variations, particularly the lower SR in the central region. Furthermore, investigating healthcare practices and access to early detection strategies in the region compared to developed nations could provide valuable insights for improving patient outcomes in Saudi Arabia.

Our study revealed consistently lower SRs across all subtypes and stages of TC compared to data from the National Cancer Institute’s SEER database and the American Cancer Society (ACS) in the United States.^8–12,14,16–22,25,26^ This disparity was particularly striking for papillary carcinoma, the most common subtype. The 5-year SR for localized papillary carcinoma in our study (96.5%) fell short of the nearly 100% rate reported in the United States (99.9%).^16,25^ This gap widened further for metastatic stages, with our study documenting a 48.4% SR for metastatic papillary carcinoma compared to the significantly higher 74.2% observed in the United States.^9^

Similar trends emerged for follicular and medullary thyroid carcinomas. According to SEER statistics, the 5-year SRs for localized, regional, and metastatic follicular thyroid carcinoma are 99.9%, 95%, and 58.6%, respectively.^11,12^ In contrast, our study found lower SRs for all stages, with localized follicular carcinoma at 92.3%, regional at 60%, and metastatic at 44.4%.^11,12^ The disparity was even more pronounced for medullary thyroid carcinoma. While the ACS reports a localized SR exceeding 99.5%, our study found a 100% rate for localized disease.^9,12^ However, this initial advantage was offset by a substantial difference in regional and metastatic SRs. The ACS reports a 92% SR for regional medullary thyroid carcinoma, compared to 75% in our study.^25^ The most concerning disparity was observed in metastatic medullary carcinoma, where no patients in our study survived beyond 5 years, contrasting with the 1% SR reported in the United States.^9^ This finding aligns with the aggressive nature of anaplastic thyroid carcinoma, the least common but most aggressive subtype. Our study documented no survivors of metastatic anaplastic thyroid carcinoma beyond 5 years, compared to the 1% SR reported in the United States.^14^

Our study revealed consistently lower SRs across all stages and subtypes of TC when compared to data from the United States (SEER and ACS).^9,25^ This significant disparity necessitates further investigation into potential contributing factors. One possibility is a higher prevalence of advanced stage disease at presentation in our Saudi Arabian patient population compared to the United States. This aligns with the well-established importance of early detection for improving TC survival outcomes.^25^ Future research should explore potential variations in access to screening programs or diagnostic modalities between the two countries. These variations might explain the observed differences in stage distribution at presentation. Additionally, investigating healthcare system variations within Saudi Arabia, such as access to advanced treatment options, could be a crucial factor and warrant further examination. Furthermore, understanding if this observed disparity is specific to SA or reflects a broader trend in the region is essential. Studies from neighboring countries in the Arabian Peninsula, like the United Arab Emirates or Qatar, could shed light on potential similarities or differences in TC management across the region.^27,28^

Our findings also confirm a well-documented gender disparity in TC survival. Women in our study exhibited a higher overall SR (90%) compared to men (82.5%). This aligns with research from other countries, including the United Kingdom (90% vs. 85%), the United States (99% vs. 96.6%), and Brazil (97.0% vs. 91.5%).^9,18,19^ This advantage for women might be attributed to the higher prevalence of the less aggressive papillary subtype diagnosed at earlier stages in this population.^20^ Finally, our study reinforces the established influence of age on TC survival. Similar to findings by Kauffmann et al., patients diagnosed at 70 or older had a significantly higher risk of death compared to younger patients (50 or younger).^23^ This risk has further increased for patients over 60 compared to those below 40. Future research should explore the reasons behind this age-related disparity to identify potential areas for intervention and improve overall SRs, particularly for older patients. Moreover, our study revealed a significant impact of the cancer subtype on SRs. Anaplastic carcinoma, the most aggressive form, demonstrated a mortality rate six times higher compared to follicular carcinoma, our chosen reference group. This aligns with previous research by Ragazzi M et al., who reported a staggering 90% mortality rate and an average lifespan of just 6 months following an anaplastic carcinoma diagnosis.^29^ This highlights the critical need for early detection and intervention in this highly aggressive subtype.

Metastatic spread also emerged as a powerful determinant of survival. Patients diagnosed with metastatic tumors faced a nearly seven times greater risk of death compared to those with localized cancer. This finding underscores the importance of identifying and treating TC at earlier stages when the potential for successful intervention is considerably higher.

## 5. Limitations

Despite its valuable insights, our study has inherent limitations due to its retrospective, single-center design relying on secondary data collected at the KAMCCR. This retrospective approach may introduce bias due to potential inconsistencies in data collection and missing information for some patients. Additionally, we were unable to account for all factors potentially influencing survival, such as tumor size, molecular markers, thyroid hormone levels, co-existing medical conditions, and specific treatment regimens employed. Future multicenter studies encompassing a broader geographical range within SA are warranted to explore these factors and elucidate any potential regional variations in presentation, treatment, and survival outcomes. Furthermore, comparative studies with neighboring countries in the Arabian Peninsula, such as the United Arab Emirates or Qatar, could provide valuable insights into potential similarities or differences in TC management across the region.

## 6. Conclusion

Our study highlights the need for a multi-pronged approach to improve TC survival rates in Saudi Arabia. Investigating potential explanations for the observed disparity compared to developed nations, exploring healthcare system variations within the region, and understanding the role of factors like gender and age are crucial steps toward achieving better patient outcomes.

### 6.1. Public health implications

Our findings underscore the critical role of early detection in improving TC survival rates, particularly for aggressive subtypes like anaplastic carcinoma. Therefore, we advocate for a public awareness campaign focusing on educating women and primary care physicians about the importance of routine thyroid gland examinations and regular thyroid hormone testing. These campaigns should be culturally sensitive and tailored to the specific needs of the Saudi population. In addition, it ensures the widespread adoption of fine-needle aspiration cytology (FNAC) as the gold standard for the initial evaluation of thyroid nodules. Moreover, the implementation of standardized FNAC protocols across healthcare facilities in SA will promote early and accurate diagnosis, ultimately leading to better patient outcomes.

By implementing these strategies, policymakers can enhance access to early detection tools and ultimately improve TC survival rates within the Kingdom. Furthermore, ongoing research efforts that explore regional variations and incorporate data from neighboring countries can provide a more comprehensive understanding of TC management across the Arabian Peninsula, paving the way for evidence-based improvements in public health strategies.

## Conflict of Interest

The authors declare they have no conflicts of interest.

## Ethics Approval

No identifiers were collected, and all the data were kept in a secure place within the College of Public Health and Health Informatics (CPHHI) premises and accessed by the research team only. The PI received a license for using the data from the General Director of School Health, MOH. The study was approved by the IRB of the King Abdullah International Medical Centre (SP19/105/R).

## Authors’ Contributions

Concepts and design: AB and SA. Data collection: SA, AB. Data and statistical analysis: AB, SA, and OA-A. Manuscript preparation, review, and editing: AB, SA, OA-A, AJ, and WK. All authors agree to the final version of the manuscript. Guarantor: AB.

## Acknowledgments

We thank King Abdulaziz Medical City Cancer Registry (KAMCCR), Riyadh, Saudi Arabia, and King Abdullah International Medical Centre for their support.

## Figures and Tables

**Table 1.** The association between the survival status of the patients and the demographic and clinicopathologic variables.

**Variables**	**Categories**	**All**	**Alive 588 (88.4%)**	**Deaths 77 (11.6%)**	***p*-value**
		**No. (%)**	No. (%)*	No. (%)*	
Gender	Male	143 (21.5)	118 (82.5)	25 (17.5)	0.013
	Female	522 (78.5)	470 (90.0)	52 (10.0)	
Age at diagnosis	<50 years	241 (36.2)	238 (98.8)	3 (1.2)	<0.001
	50-59 years	167 (25.1)	158 (94.6)	9 (5.4)	
	60-69 years	124 (18.6)	106 (85.5)	18 (14.5)	
	≥70 years	133 (20.0)	86 (64.7)	47 (35.3)	
Stage	Localized	346 (53.7)	332 (96.0)	14 (4.0)	<0.001
	Regional	243 (37.7)	217 (89.3)	26 (10.7)	
	Distant	55 (8.5)	20 (36.4)	35 (63.6)	
Histopathologic	PTC^†^	589 (88.6)	542 (92.0)	47 (8.0)	<0.001
subtype	FTC^‡^	46 (6.9)	35 (76.1)	11 (23.9)	
	MTC^§^	11 (1.7)	10 (90.9)	1 (9.1)	
	ATC^II^	19 (2.9)	1 (5.3)	18 (94.7)	

* Calculated by rows.

^†^ PTC-papillary thyroid carcinoma,

^‡^ FTC-Follicular thyroid carcinoma,

^§^ MTC-medullary thyroid carcinoma,

^II^ ATC-anaplastic thyroid carcinoma.

**Table 2.** The association between the survival status of the patients and the stage of the cancer.

**Variables**	**Categories**	**Alive 588 (88.4%)**	**Deaths 77(11.6%)**	***p*-value**
**Stage**	**Morphology**	**No. (%)**	No. (%)*	
Localized	PTC^†^	301(96.5)	H (3.5)	<0.001
	FTC^‡^	24 (92.3)	2 (7.7)	
	MTC^§^	7 (100.0)	0 (0.0)	
	ATC^II^	0 (0.0)	1(100.0)	
Regional	PTC^†^	208 (92.0)	18 (8.0)	<0.001
	FTC^‡^	6 (60.0)	4 (40.0)	
	MTC^§^	3 (75.0)	1(25.0)	
	ATC^II^	0 (0.0)	3 (100.0)	
Distant	PTC^†^	15 (48.4)	16 (51.6)	0.019
	FTC^‡^	4 (44.4)	5 (55.6)	
	MTC^§^	0 (0.0)	0 (0.0)	
	ATC^II^	1(6.7)	14 (93.3)	

* Calculated by rows.

^†^ PTC-papillary thyroid carcinoma,

^‡^ FTC-Follicular thyroid carcinoma,

^§^ MTC-medullary thyroid carcinoma,

^II^ ATC-anaplastic thyroid carcinoma.

**Table 3.** Hazard ratio concerning demographics and clinicopathologic characteristics of the TC patients.

**Variables**	**Categories**	HR*	**95% CI**	***p*-value**	Adjusted HR*	**95% CI**	***p*-value**
Gender	Male	-	-	-	-	-	-
	Female	1.087	0.658–1.796	0.745	-	-	-
Age at diagnosis	<50 years	Reference	-	-	Reference	-	-
	50–59 years	4.302	1.135–16.307	0.032	4.301	1.135–16.304	0.032
	60–69 years	11.664	3.387–40.164	<0.001	11.520	3.352–39.597	<0.001
	≥70 years	18.630	5.599–61.988	<0.001	18.351	5.535–60.846	<0.001
Stage	Localized	Reference	-	-	Reference		-
	Regional	2.76 7	1.428–5.363	0.003	2.736	1.416–5.287	0.003
	Distant	7.763	3.707–16.256	<0.001	7.692	3.682–16.069	<0.001
Histopathologic	FTC^†^	Reference	-	-	Reference		-
subtype	PTC^‡^	0.661	0.326–1.341	0.251	0.666	0.329–1.348	0.259
	MTC^§^	1.200	0.148–9.736	0.864	1.166	0.145–9.384	0.885
	ATC^II^	6.856	2.841–16.547	<0.001	6.834	2.833–16.481	<0.001

* HR-hazard ratio,

^†^ FTC-papillary thyroid carcinoma,

^‡^ PTC-Follicular thyroid carcinoma,

^§^ MTC-medullary thyroid carcinoma,

^II^ ATC-anaplastic thyroid carcinoma.
